# MOG-IgG in NMO and related disorders: a multicenter study of 50 patients. Part 3: Brainstem involvement - frequency, presentation and outcome

**DOI:** 10.1186/s12974-016-0719-z

**Published:** 2016-11-01

**Authors:** Sven Jarius, Ingo Kleiter, Klemens Ruprecht, Nasrin Asgari, Kalliopi Pitarokoili, Nadja Borisow, Martin W. Hümmert, Corinna Trebst, Florence Pache, Alexander Winkelmann, Lena-Alexandra Beume, Marius Ringelstein, Oliver Stich, Orhan Aktas, Mirjam Korporal-Kuhnke, Alexander Schwarz, Carsten Lukas, Jürgen Haas, Kai Fechner, Mathias Buttmann, Judith Bellmann-Strobl, Hanna Zimmermann, Alexander U. Brandt, Diego Franciotta, Kathrin Schanda, Friedemann Paul, Markus Reindl, Brigitte Wildemann, in cooperation with the Neuromyelitis Optica Study Group (NEMOS)

**Affiliations:** 1 Molecular Neuroimmunology Group, Department of Neurology, University Hospital Heidelberg, Otto Meyerhof Center, Im Neuenheimer Feld 350, 69120 Heidelberg, Germany; 2 Department of Neurology, Ruhr University Bochum, Bochum, Germany; 3 Department of Neurology, Charité – University Medicine Berlin, Berlin, Germany; 4 Department of Neurology and Institute of Molecular Medicine, University of Southern Denmark, Odense, Denmark; 5 Department of Neurology, Hannover Medical School, Hannover, Germany; 6 Department of Neurology, NeuroCure Clinical Research Center and Clinical and Experimental Multiple Sclerosis Research Center, Charité University Medicine, Berlin, Germany; 7 Experimental and Clinical Research Center, Max Delbrueck Center for Molecular Medicine, Berlin, Germany; 8 Department of Neurology, University of Rostock, Rostock, Germany; 9 Department of Neurology, Albert Ludwigs University, Freiburg, Germany; 10 Department of Neurology, Heinrich Heine University, Düsseldorf, Germany; 11 Department of Neuroradiology, Ruhr University Bochum, Bochum, Germany; 12 Institute of Experimental Immunology, affiliated to Euroimmun AG, Lübeck, Germany; 13 Department of Neurology, Julius Maximilians University, Würzburg, Germany; 14 IRCCS, C. Mondino National Neurological Institute, Pavia, Italy; 15 Department of Neurology, Medical University Innsbruck, Innsbruck, Austria

**Keywords:** Myelin oligodendrocyte glycoprotein (MOG) antibodies, MOG-IgG, Neuromyelitis optica spectrum disorders (NMOSD), Brainstem encephalitis, Rhombencephalitis, Optic neuritis, Myelitis, Longitudinally extensive transverse myelitis (LETM), Cerebellitis, Ataxia, Respiratory insufficiency, Intractable nausea and vomiting﻿, Facial nerve palsy, Diplopia Internuclear ophthalmoplegia (INO), Hearing loss, Aquaporin-4 antibodies (AQP4-IgG, NMO-IgG)

## Abstract

**Background:**

Myelin oligodendrocyte glycoprotein antibodies (MOG-IgG) are present in a subset of aquaporin-4 (AQP4)-IgG-negative patients with optic neuritis (ON) and/or myelitis. Little is known so far about brainstem involvement in MOG-IgG-positive patients.

**Objective:**

To investigate the frequency, clinical and paraclinical features, course, outcome, and prognostic implications of brainstem involvement in MOG-IgG-positive ON and/or myelitis.

**Methods:**

Retrospective case study.

**Results:**

Among 50 patients with MOG-IgG-positive ON and/or myelitis, 15 (30 %) with a history of brainstem encephalitis were identified. All were negative for AQP4-IgG. Symptoms included respiratory insufficiency, intractable nausea and vomiting (INV), dysarthria, dysphagia, impaired cough reflex, oculomotor nerve palsy and diplopia, nystagmus, internuclear ophthalmoplegia (INO), facial nerve paresis, trigeminal hypesthesia/dysesthesia, vertigo, hearing loss, balance difficulties, and gait and limb ataxia; brainstem involvement was asymptomatic in three cases. Brainstem inflammation was already present at or very shortly after disease onset in 7/15 (47 %) patients. 16/21 (76.2 %) brainstem attacks were accompanied by acute myelitis and/or ON. Lesions were located in the pons (11/13), medulla oblongata (8/14), mesencephalon (cerebral peduncles; 2/14), and cerebellar peduncles (5/14), were adjacent to the fourth ventricle in 2/12, and periaqueductal in 1/12; some had concomitant diencephalic (2/13) or cerebellar lesions (1/14). MRI or laboratory signs of blood-brain barrier damage were present in 5/12. Cerebrospinal fluid pleocytosis was found in 11/14 cases, with neutrophils in 7/11 (3-34 % of all CSF white blood cells), and oligoclonal bands in 4/14. Attacks were preceded by acute infection or vaccination in 5/15 (33.3 %). A history of teratoma was noted in one case. The disease followed a relapsing course in 13/15 (87 %); the brainstem was involved more than once in 6. Immunosuppression was not always effective in preventing relapses. Interferon-beta was followed by new attacks in two patients. While one patient died from central hypoventilation, partial or complete recovery was achieved in the remainder following treatment with high-dose steroids and/or plasma exchange. Brainstem involvement was associated with a more aggressive general disease course (higher relapse rate, more myelitis attacks, more frequently supratentorial brain lesions, worse EDSS at last follow-up).

**Conclusions:**

Brainstem involvement is present in around one third of MOG-IgG-positive patients with ON and/or myelitis. Clinical manifestations are diverse and may include symptoms typically seen in AQP4-IgG-positive neuromyelitis optica, such as INV and respiratory insufficiency, or in multiple sclerosis, such as INO. As MOG-IgG-positive brainstem encephalitis may take a serious or even fatal course, particular attention should be paid to signs or symptoms of additional brainstem involvement in patients presenting with MOG-IgG-positive ON and/or myelitis.

## Background

Over the past few years, a new diagnostic role has been found for antibodies to myelin oligodendrocyte glycoprotein (MOG-IgG) in adults [1]. While MOG-IgG had initially been thought to play a role in classical multiple sclerosis (MS), recent studies have demonstrated that MOG-IgG are in fact a marker of autoimmune optic neuritis (ON) and (often longitudinally extensive) transverse myelitis [1, 2]. Based on the fact that aquaporin-4 (AQP4)-IgG is usually absent in MOG-IgG-positive patients [3–10], that the histopathology of inflammatory CNS lesions differs between MOG-IgG- and AQP4-IgG-positive patients [11–13], and that MOG-IgG are pathogenic both in vitro and in vivo [2, 14], MOG-IgG-related autoimmunity is now considered by many a disease entity in its own right, distinct both from classical MS and from AQP4-IgG-mediated neuromyelitis optica spectrum disorders (NMOSD) [15, 16].

While the association of MOG-IgG with ON and myelitis is now well established [2–4, 6, 17], less is known about extra-opticospinal manifestations in MOG-IgG-related autoimmunity. Here, we report the largest series of Caucasian patients with MOG-IgG-positive brainstem encephalitis so far. Brainstem involvement was severe in some of the cases and was fatal in one patient. Clinical, laboratory, and radiologic findings are reported in addition to treatment outcomes.

This article is the third of a four-part series on the clinical, laboratory, magnetic resonance imaging (MRI), electrophysiological, and optical coherence tomography features of patients with MOG-IgG-related CNS autoimmunity [3, 17, 18].

## Methods

All 15 patients were identified from a large European cohort of almost exclusively Caucasian patients with MOG-IgG-associated ON and/or myelitis (*n* = 50) from 12 European academic centers, eight of which are members of the German Neuromyelitis optica study group (NEMOS) [19–23]; this cohort is described in parts 1 and 2 of this series [3, 17]. MOG-IgG had been tested for clinical purposes in all cases and was detected using a live-cell-based assay (CBA) [2] and a commercial fixed-cell CBA (Euroimmun, Luebeck, Germany), both of which employ recombinant human full-length MOG as antigenic substrate. AQP4-IgG was tested using a commercial CBA (Euroimmun) employing recombinant human full-length AQP4 [24–26] and was negative in all cases [3]. The study was approved by the institutional review boards of the participating centers. Patients gave their informed consent for publication. The median number of documented clinically apparent brainstem attacks per patient was 1 (range 1-5); brainstem encephalitis was asymptomatic in three patients. In total, 27 brainstem events were analyzed, including 21 symptomatic brainstem attacks and 6 ON and/or myelitis attacks that were associated with MRI evidence for concomitant subclinical brainstem encephalitis. In 6 patients, the brainstem was involved more than once. The patients’ median disease duration at last follow-up was 54 months (range 10-507; over >24 months in 12/15 cases).

## Case reports

**Fig. 1:**
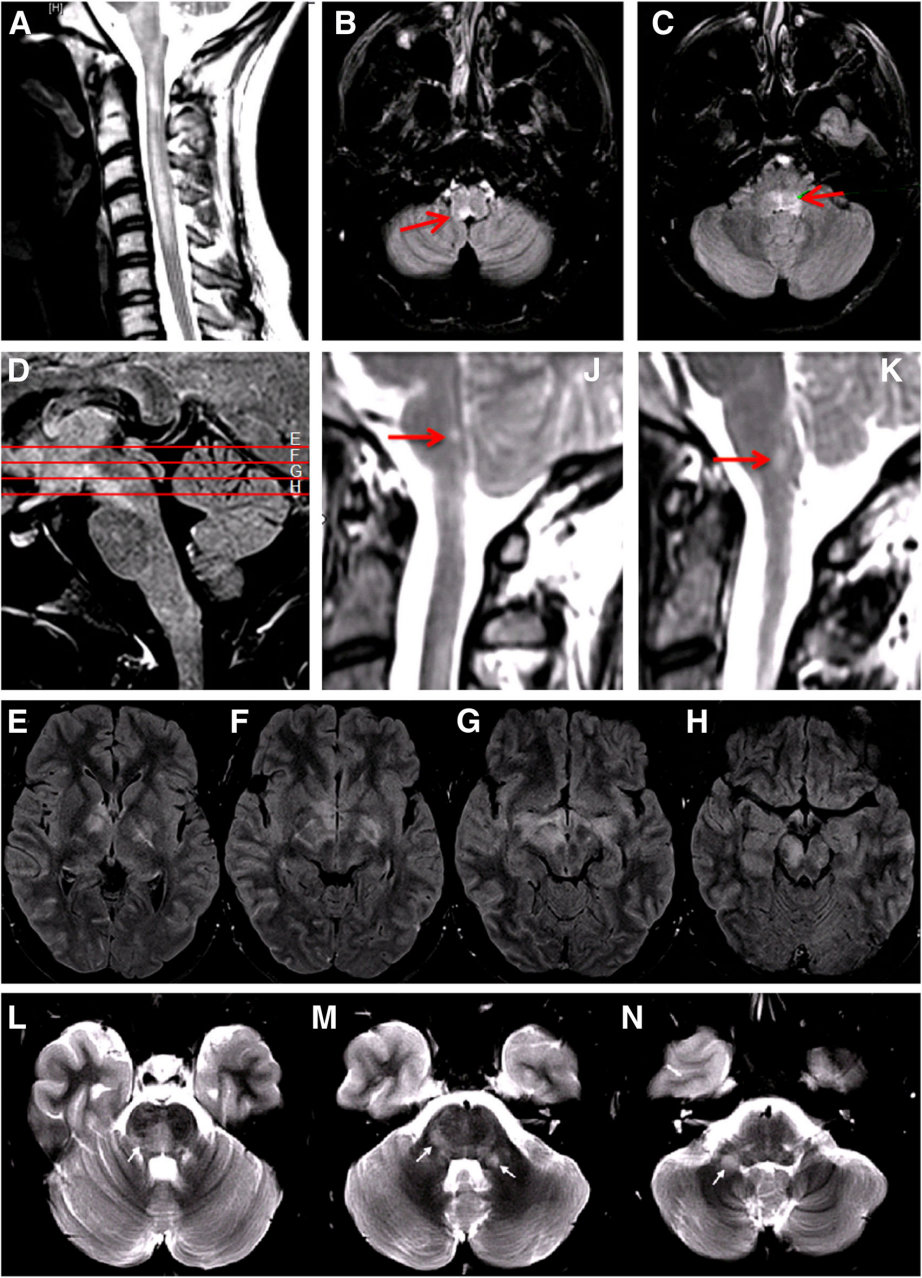
Examples of the magnetic resonance imaging (MRI) findings in patients with MOG-IgG-positive brainstem encephalitis. **a**-**c** Patient 12: T2-hyperintense lesions extending from the pontomedullary junction throughout the cervical cord as far as C5; lesions included the dorsal medulla oblongata (B, *arrow*). **d**-**h** Patient 8: T2-hyperintense lesions in the pons, midbrain, thalamus, and basal ganglia; lesions involved the periependymal surfaces of the third ventricle. **j**-**k** Patient 4: T2-hyperintense lesions in the right (**j**) and left (**k**) half of the dorsal medulla oblongata including the area postrema (**j**). **l**-**n** Patient 2: T2-hyperintense lesions in the frontal and parietal subcortical white matter, the pontine tegmentum, and the cerebellar peduncles (*arrows*)

**Fig. 2:**
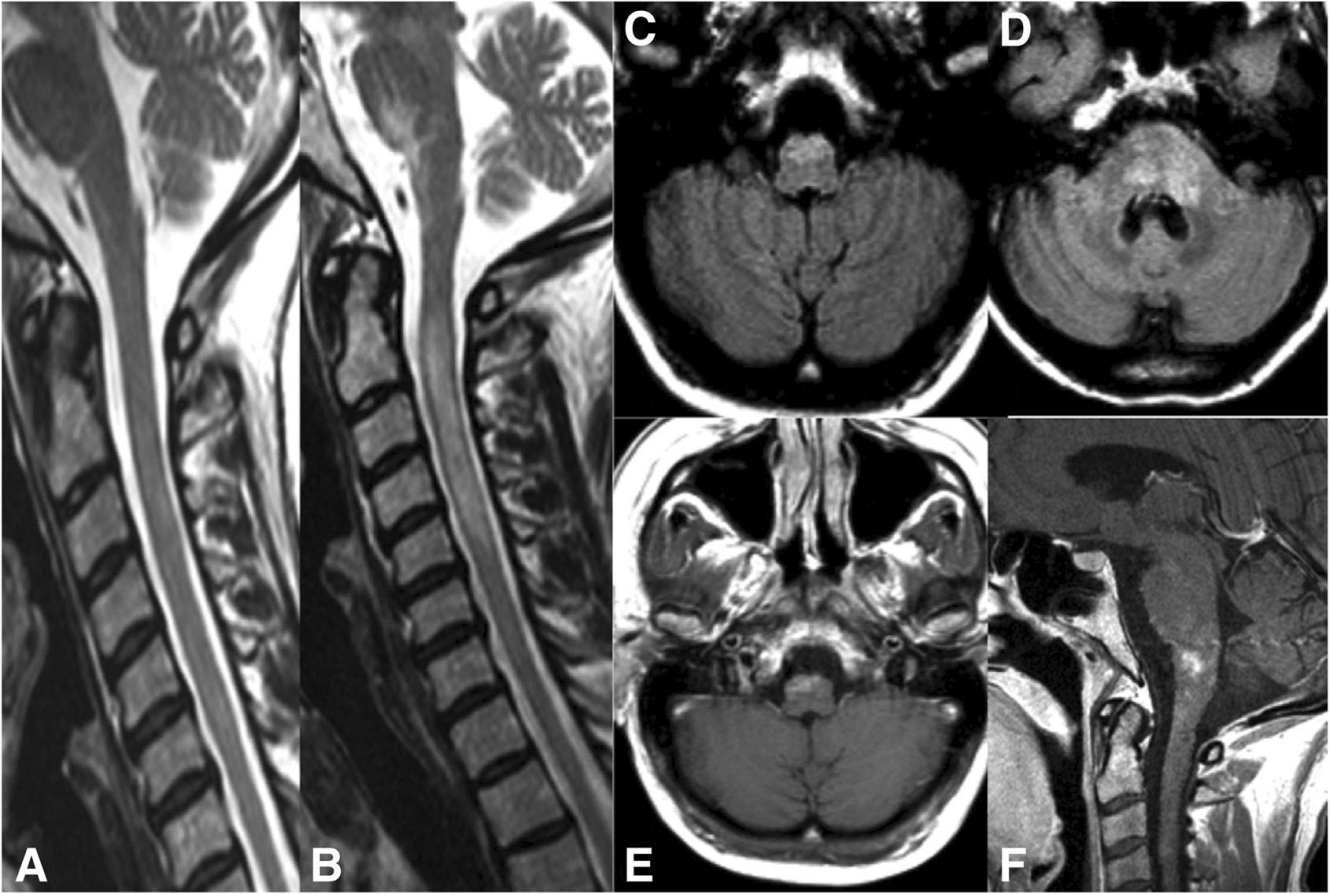
Serial MRI examination of patient 1. **a** Sagittal T2 weighted baseline MRI showing no involvement of the brainstem or of the spinal cord. **b-e** Follow-up MRI 5 month later revealed a new spinal cord lesion extending over 4 vertebral segments with cord swelling on T2-weighted imaging as well as new lesion formation in the pons and medulla oblongata with gadolinium enhancement on T1-weighted imaging. Within the following 5 months clinical symptoms deteriorated further with infratentorial T2 lesion enlargement and new lesion formation in the pons, e.g. adjacent to the middle cerebellar peduncle, accompanied by gadolinium enhancement (**f**)

As reliable tests for MOG-IgG have only recently become available, comprehensive case series illustrating the broad and heterogeneous spectrum of clinical manifestations, disease courses, and radiologic presentations in MOG-IgG-positive patients are lacking so far. In particular, there are almost no detailed reports on patients with brainstem encephalitis. To paint for the first time a vivid ‘real-life’ picture of the disorder, which statistical analyses alone cannot achieve, we decided to provide detailed reports on all patients included in this study. Cases 1, 2 and 3 are described below; reports on the remaining 12 cases are to be found in the Appendix. For a comprehensive summary of the patients’ clinical, radiological and laboratory features see the Table 1. A selection of illustrative MR images demonstrating brainstem damage in MOG-IgG-positive patients is shown in Figs. 1 and 2.

**Table 1:** Clinical, radiological and laboratory findings in 15 MOG-IgG-positive patients with a history of brainstem involvement

	#1	#2	#3	#4	#5	#6	#7	#8	#9	#10	#11	#12	#13	#14	#15
Sex	Ff	f	f	f	f	f	m	m	m	m	f	f	f	f	f
Ethnicity	Cauc	Cauc	Cauc	Cauc	Cauc	Cauc	Cauc	Cauc	Cauc	Cauc	Cauc	Cauc	Cauc	Cauc	Cauc
BSTI at onset	y	y	n	n	n	n	n	n	y	y	y	y	n	y	n
Age at first evidence for BSTI (years)	53	18	45	35	31	53	19	19	50	37	44	27	26	25	22
Time to first evidence for BSTI (years)	0	0	18	0.75	0.25	41	0.3	0.17	0	0	0	0	1.7	0	0
No of clinical BST attacks	5	1	2	3	1	0	1	1	0	1	1	1	1	2	1
No of attacks with subclinical BSTI	2	0	0	0	0	1	0	1	1	0	0	0	1	0	0
Clinical BST findings	Central hypoventilation, dysphagia, dysarthria, CN III and VII paresis	Respiratory impairment, difficulties coughing, dysphagia, dysarthria, diplopia	Cerebellar gait and upper limb ataxia	Impaired balance, vertigo	Intractable nausea and vomiting	None, subclinical BST involvement	Double vision and gait ataxia	Hearing loss	None, subclinical BST involvement	Trigeminal hypesthesia	None, subclinical BST involvement	Trigeminal hyp- and paresthesia, diplopia, nystagmus, unsteady gait	Hypesthesia tongue and face, impaired smooth pursuit	Hemihypesthesia including the face	INO
Infratentorial MRI findings	MRI1: Cerebral peduncle of the midbrain, MO, pons, MRI2: MO, new T2 lesions, Gd+, MRI3: pedunculus cerebellaris, crus cerebri, patchy Gd + pons, MO	Pontine tegmentum and cerebellar peduncles	Crus cerebri and entire pons, around the 4^th^ ventricle, extending into the left cerebellar hemisphere	Pons and medulla oblongata	Right and left dorsal MO, ad-jacent to the 4^th^ ventricle, incl. the area postrema	MO with patchy Gd enhancement	Lesions in the peri-aqueductal gray, ventral pons	T2 lesion in the pons	Median pontine lesion	N.d.	Cerebellar peduncle, single lesion	T2 lesions extending from the ponto-med. junction, throughout the MO to C5, incl. around the 4^th^ ventricle	T2 lesions in the MO and pons, detectable over a period of at least 12 months	Bilateral pontine lesions and bilateral cerebellar peduncle lesions	Large, Gd + lesion: pons bilat., both pedunculi cerebelli, paramedian ponto-medullary junction
Cerebral peduncles	y	n	y	n	n	n	n	n	n	n.d.	n	n	n	n	n
Pons	y	y	y	y	n.d.	n	y	y	y	n.d.	n	y	y	y	y
Cerebellar peduncles	y	y	n	n	n	n	n	n	n	n.d.	y	n	n	y	y
Cerebellum	n	n	y	n	n	n	n	n	n	n.d.	n	n	n	n	n
Medulla oblongata	y	n	y	y	y	y	n	n	n	n.d.	n	y	y	n	y
Bulbo-spinal lesion, ever	y	n	n	y	y	y	n	n	n	n.d.	n	y	n	n	n
Gd+, ever	y	n	y	y	y	y	y	y	y	n.d.	n.d.	n	n	n.d.	y
Supratentorial MRI findings	Normal	T2-hyperintense lesions in the frontal and parietal subcortical white matter	Crus cerebri, left subcort. white matter (adjacent to the temporal horn), corpus callosum, juxtacortical regions of parietal lobes	Lateral ventricular lesions	Normal	Normal	Confluent T2 hyperintense lesions in the right temporal lobe, pulvinar bilaterally	T2 lesions in basal ganglia, corpus callosum, periventr., pulvinar thalami, rostral putamen; leptomeningeal contrast enhancement	Normal	Normal	Single frontal lobe lesion	Juxta-cortical T2 lesion, insular region	Callosal, periventr., juxtacortical, deep white matter	Single small lesion directly adjacent to the left lateral ventricle	Peritrigonally and corona radiate, Gd+
Postinfectious/postvaccinal	n	y	n	n	n	y	n	y	y	n	n.d.	n	n	y	n
Simultaneous ON and BSTI	y	n	n	n	n	n	n	y	y	y	n	y	n	n	n
Simultaneous MY and BSTI	y	y	n	y	y	y	y	y	y	y	y	y	y	n	y
History of both ON and MY	y	n	n	y	y	y	y	y	y	y	y	y	y	n	y
Recurrent disease	y	n	y	y	y	y	y	y	y	n	y	y	y	y	y
NMOSD 2015	n	n	y	y	y	n	y	y	n	n	y	y	y	n	y
CSF-restr. OCB	n	n	n	y	n.d.	n	y	y	n	n	n	n	y	n	n
CSF WCC	normal	360	50	normal	122	30	22	60	normal	8	n.d.	59	33	150	8
CSF neutrohils	n.a.	7 %	6 %	n.a.	3 %	n.d.	6 %	26 %	n.a.	34 %	n.d.	n.a.	n.d.	26 %	n.d.
QAlb elevated	n.d.	y	n.d.	n	n	y	n	y	n	n	n.d.	n	n	y	y
Last EDSS	10	1	7.5	3	3	4.5	1	0	1	0	2.5	0	3.5	3	0.5

*Abbreviations*: *BSTI* brainstem involvement, *y* yes, *n* no, *n.a.* not applicable, *n.d.* no data, *f* female, *m* male, *Cauc* Caucasian, *MO* medulla oblongata, *Gd +* gadolinum enhancing, *CN* cranial nerve, *MRI* magnetic resonance imaging, *MY* myelitis, *ON* optic neuritis, *NMOSD 2015* neuromyelitis optica spectrum disorder according to Wingerchuk et al. (2015), *CSF* cerebrospinal fluid, *OCB* oligoclonal bands, *QAlb* albumin CSF/serum ratio, *EDSS* extended disability status scale, *INO* internuclear ophthalmoplegia

### Case 1 – Fatal rhombencephalitis in a patient with recurrent ON and LETM

A previously healthy 44-year-old Caucasian woman first developed right-sided ON in November 2003. Lumbar puncture (LP) demonstrated a normal CSF white cell count as well as normal CSF protein, glucose, and lactate levels and negative oligoclonal bands (OCB). Brain MRI revealed asymptomatic lesions in the right cerebellar hemisphere and in the cerebral peduncle of the midbrain. Magnetic resonance imaging (MRI) of the spinal cord was not performed. After treatment with intravenous methylprednisolone (IVMP), vision returned to normal. Extensive laboratory tests did not reveal any infectious or rheumatologic cause of the patient’s symptoms.

Up to 2010 ON attacks occurred once to twice annually, but vision always returned to normal after IVMP therapy. However, after another attack of ON in August 2010, vision in the right eye dropped permanently to 0.85 on the Snellen chart despite IVMP therapy.

In January 2011, the patient developed a first attack of myelitis with accompanying brainstem encephalitis. Symptoms included right hemiparesis, dysarthria, and dysphagia. MRI showed no supratentorial brain lesions but a T2 lesion in the medulla oblongata and a cervical longitudinally extensive transverse myelitis (LETM) lesion extending from C2 to C4 with gadolinium (Gd) enhancement. LP again demonstrated a normal CSF white cell count, normal CSF protein level, and negative OCB. Treatment with IVMP was followed by complete remission. In June 2011, after another attack of myelitis with paraparesis, treatment of which with IVMP had resulted in incomplete remission of the symptoms, treatment with glatiramer acetate (GLAT) for suspected MS was initiated.

After a relapse-free period of 1 year, two further relapses (ON in May 2012 and myelitis with paraparesis in July 2012) followed. While treatment with IVMP led only to partial recovery, plasma exchange (PEX) treatment (five exchanges) was followed by complete clinical remission after both relapses. After the second relapse, GLAT was stopped (July 2012) and natalizumab started. However, treatment with natalizumab was discontinued after two infusions due to recurrent headaches.

In September, October, and December 2012 three further relapses of myelitis with paraparesis occurred. PEX (five exchanges; no IVMP) resulted in partial recovery in all cases. In January 2013, the patient received a first infusion of mitoxantrone (12 mg/m^2^, nadir 3000/μl), and after a further myelitis relapse with paraparesis in February 2013, a second mitoxantrone infusion was given (8 mg/m^2^, nadir 2800/μl). Complete recovery was achieved following PEX (five exchanges; no IVMP). After a further flare-up of myelitis later the same month with paraparesis and Gd enhancement at levels T2, T3, T5, and T9, treated with a cycle of five plasmaphereses with incomplete remission in February 2013, a third infusion of mitoxantrone with 8 mg/m^2^ was given. A serum sample taken at that time later tested positive for MOG-IgG in a live-cell CBA [2] (1:1280). Early in 2013, two new ON relapses occurred and were treated with a cycle of PEX (five exchanges), again with incomplete remission. MRI now showed Gd enhancement in large parts of the thoracic spinal cord. An expanded disability status scale (EDSS) score of 6 was documented at that time. MOG-IgG were retrospectively positive at a titer of 1:640.

Rituximab (500 mg) was infused for the first time in May 2013, followed 2 weeks later by a second infusion of 500 mg rituximab. Fifteen days after the second infusion the patient’s paraparesis worsened (EDSS 8) and dysarthria re-occurred. Cerebral and spinal MRI showed active lesions with Gd enhancement at the C4/C5 level, in the pons, and in the medulla oblongata. After PEX (five exchanges; no IVMP), the patient was able to walk again and her dysarthria completely remitted. Two new myelitis relapses in July and August 2013 with paraparesis (EDSS 7.5) and dysarthria were again treated with PEX (five exchanges; no IVMP), which was followed by partial recovery. MRI showed a new inactive lesion in the pons in July 2013 and in August 2013 Gd enhancement extended from C2 to C5. After a second PEX cycle, urosepsis occurred and was treated successfully with antibiotics. The next relapse occurred in November 2013 with a left ON (visual acuity of 0.05), which remitted incompletely after one cycle of PEX (five exchanges) (EDSS 7.5).

In January 2014, massive deterioration of the brainstem symptoms occurred, with dysarthria, dysphagia, left oculomotor and facial nerve palsy, and new T2-hyperintense lesions in the pons, pedunculus cerebellaris, cerebral crus, and medulla oblongata. No supratentorial lesions were seen on brain MRI. Spinal MRI demonstrated a diffuse, asymptomatic T2-hyperintense lesion from C2 to C4 as well as patchy Gd enhancement in the pons, medulla oblongata, and the entire cervical spinal cord. The patient was treated with two courses of five PEX each (no IVMP) and initially improved. In the following month, however, clinical symptoms deteriorated again and the patient developed bilateral blindness and central hypoventilation. She was transferred to palliative care and died in May 2014.

The total number of attacks in this patient was 25, 15 of which occurred under various immunomodulatory and immunosuppressive therapies. These included around 13 relapses of ON and 12 of myelitis; the brainstem was clinically affected during 5 attacks. This corresponds to an annualized relapse rate of 2.38, though 15/25 relapses occurred during the last 24 months before the patient’s death.

### Case 2 – Single episode of post-infectious whole-spine myelitis with severe brainstem and brain involvement yet complete recovery

An 18-year-old Caucasian woman had purulent tonsillitis in June 2010 and was treated with amoxicillin for 5 days. About 1 week later she developed intermittent fever (up to 39 °C), general malaise, headaches, meningism, fluctuating diplopia on right gaze, and subtle psychomotor slowing, for which she was admitted to the Department of Neurology, Charité – University Medicine Berlin. The day after admission, her condition deteriorated and she additionally noted weakness of her legs, urinary incontinence, respiratory impairment with difficulty in coughing, and mild dysphagia. On neurologic examination the patient was awake and oriented. Cranial nerve examination showed no abnormalities, but she had marked dysarthria and reported fluctuating horizontal diplopia. Her legs were plegic and she had reduced pain and touch sensation below T10. There was also British Medical Research Council (BMRC) grade 4 distal weakness in both arms. Deep tendon reflexes were preserved throughout. Except for a mature cystic ovarian teratoma without signs of malignancy, which had been removed 2 months before, her past medical history was unremarkable. N-Methyl-D-aspartate receptor (NMDAR) antibodies were negative. Spinal MRI demonstrated a prominent, longitudinally extensive, T2-hyperintense and centrally located lesion without contrast enhancement extending over almost the entire spinal cord, as well as swelling of the cord (Fig. 2). Cranial MRI showed T2-hyperintense lesions in the frontal and parietal subcortical white matter, the pontine tegmentum, and the cerebellar peduncles without contrast enhancement (Fig. 1). Thoracic and abdominal MRI revealed a right retroperitoneal mass with a diameter of 5.6 cm, which was resected 5 months later and found to be a ganglioneuroma. CSF analysis on the day of admission demonstrated an elevated total cell count with 306 white blood cells/μl (reference range <5/μl) with 80.2 % lymphocytes, 8.2 % monocytes, 7 % neutrophils, 2.4 % eosinophils, and 2.2 % activated lymphocytes, but no plasma cells or tumor cells. CSF lactate was elevated at 28.4 mg/dl (reference range <20 mg/dl). There was moderate blood--CSF barrier (BCSFB) dysfunction with an albumin CSF/serum ratio (QAlb) of 30.6 × 10^-3^ (age-adjusted upper limit of normal 5.2 × 10^-3^). Accordingly, total CSF protein was elevated (213 mg/dl; reference range <45 mg/dl). She had no local IgG synthesis and no CSF-specific OCB.

Routine serum chemistry including C-reactive protein (CRP) showed normal results. A complete blood count revealed mild leukocytosis (12.44/nl; reference range <11/nl). Extensive microbiological (herpes simplex virus type 1 and 2, varicella zoster virus, Epstein--Barr virus, cytomegalovirus, human herpes virus 6, enteroviruses, influenza A virus, adenovirus, tick-borne encephalitis virus, human immunodeficiency virus, human T-cell leukemia virus type 1, *Borrelia*, *Treponema pallidum*, *Brucella*, *Bartonella*, *Mycoplasma*, *Chlamydia*, *Mycobacterium tuberculosis*, *Aspergillus*) work-up detected no signs of acute CNS infection.

Antinuclear antibodies were detectable at a titer of 1:1280, but antibodies to double-stranded DNA, extractable nuclear antigens, onconeuronal antigens (Hu, Yo, Ri, CV2/CRMP5, Ma2/Ta, amphiphysin), cardiolipin, β2-glycoprotein, phosphatidyl serine, gangliosides (GD1a, GD1b GM1, GM2, GM3, GQ1b, GT1b [IgG and IgM in each case]), and aquaporin-4 were negative, as were anti-neutrophil cytoplasmic antibodies. Screening of serum and CSF for antibodies against NMDA receptors (IgG, IgA, IgM), alpha-amino-3-hydroxy-5-methyl-4-isoxazolepropionic acid (AMPA) receptors, glycine receptor, myelin-associated antigen, and glutamic acid decarboxylase was likewise negative.

However, testing of a serum sample obtained on the day of admission in a live-cell CBA demonstrated high-titer serum IgG antibodies against MOG (1:10240). Anti-MOG antibodies were also detectable in the CSF (1:64), but there was no evidence of intrathecal production of anti-MOG antibodies, with an anti-MOG antibody index (AI) of 0.3 (reference range <4) [3].

The patient was initially treated with ceftriaxone and ampicillin, which was discontinued after a bacterial infection was ruled out, as well as with IVMP (1 g/day for 6 days). Corticosteroids were thereafter administered orally (prednisolone 100 mg/d), slowly tapered, and eventually stopped in October 2010. Starting on the day after admission, she was additionally treated with a total of 10 PEX. MOG-IgG was retrospectively detectable also in PEX plasma (titer 1:320 after three exchanges). Thereafter, therapy with intravenous immunoglobulins (IVIG, 2 g/kg body weight) was initiated in July 2010. IVIG therapy was continued at 5-week intervals until June 2011, with dose reduction to 1 g/kg body weight from January 2011 onwards. The patient gradually recovered and was able to walk with a walking frame by August 2010. Follow-up spinal MRI in August 2010 demonstrated resolution of the longitudinal extensive lesion, but there were several residual patchy hyperintense lesions within the spinal cord. In a serum sample obtained at the same time, the anti-MOG antibody titer was clearly lower (1:640) than the initial titer, and anti-MOG antibodies were no longer detectable in CSF.

The patient did not receive any further immunomodulatory or immunosuppressive therapy. At the last follow-up examination, 28 months after disease onset and 16 months after discontinuation of IVIG, she reported no further relapses and had no impairments in activities of daily living except for residual unsteadiness when walking for a longer time, difficulties on running for more than 5 min, and slightly increased urinary frequency (EDSS 1). Neurologic examination showed a very mild residual right-sided pyramidal syndrome with brisk tendon reflexes, but was otherwise unremarkable.

### Case 3 – Recurrent ON with rhombencephalitis and extensive brain involvement with poor outcome

A Caucasian woman experienced a first episode of bilateral ON in 1985 at age 28 and had three further attacks of bilateral and two of unilateral ON up to 1999, leaving her with residual visual acuity of <0.2 in both eyes. CSF and brain MRI was normal. In October 2003, the patient presented with intractable headaches lasting for several days, followed by mild impairment of consciousness. Cerebral computed tomography showed no abnormalities, while CSF analysis disclosed mixed lymphocytic and neutrophilic pleocytosis (1024 cells/μl) along with increased total protein (1350 mg/l) and mildly elevated lactate (3.6 mmol/l). CRP and leukocytes were elevated at 18 mg/l and 20.9 Gpt/l, respectively. There was no evidence of an infectious etiology, and symptoms resolved after combined treatment with dexamethasone 10 mg 4× per day for 4 days and ceftriaxone 4 g daily for 10 days. However, within 2 weeks the patient developed bilateral intention tremor of the upper extremities, marked gait ataxia, and right-sided visual worsening. Cranial MRI revealed extensive T2 hyperintense lesions in the brainstem, including around the fourth ventricle and in the supratentorial white matter, the corpus callosum, and, to some extent, the cerebral cortex. None of these abnormalities showed Gd enhancement. The CSF had 50 white cells/μl, mostly lymphocytes with some neutrophils (6 %) and occasional eosinophils (2 %). Treatment with IVMP (1 g/d for 5 days) followed by oral tapering of steroids for 6 weeks resulted in marked improvement of symptoms.

Six months later, the patient noted visual worsening in the left eye and recurrence of gait ataxia. Repeat cranial MRI depicted progressive T2-hyperintense lesions in the entire pons, especially around the fourth ventricle and the foramina of Luschka, extending bilaterally into the white matter of the left cerebellar hemisphere and into the crus cerebri with Gd enhancement. There was also involvement of the left subcortical white matter (adjacent to the temporal horn), the corpus callosum, and the juxtacortical regions of both parietal lobes, along with contrast enhancement in some of these lesions. Cerebral angiography showed no abnormalities. CSF analysis showed 5 cells/μl with a predominance of lymphocytes and some neutrophils and eosinophils. There was no evidence of intrathecal immunoglobulin synthesis. Anti-Hu and anti-Yo antibodies were negative. The patient received 1000 mg methylprednisolone i.v. daily for 5 days and subsequently long-term oral steroids, and this coincided with partial amelioration of both clinical symptoms and radiologic findings. Nightmares and visual hallucinations resolved along with rapid tapering of steroid doses. For long-term immunosuppression, overlapping treatment with azathioprine (AZA) (150 mg/day) was initiated in August 2004. However, five more relapses occurred over the following 101 months despite AZA treatment. At last follow-up, the EDSS of 7.5 and bilateral optic nerve atrophy (involving the chiasm) was documented. Retrospective testing of a serum sample taken in 2010 revealed high-titer MOG-IgG (1:20,480). MOG-IgG seropositivity was confirmed in a second sample obtained 22 months later.

## Discussion

We describe the largest series to date of MOG-IgG-positive patients with brainstem involvement, thereby expanding the clinical spectrum of MOG-IgG autoimmunity. The 15 cases presented here were identified as part of a large European cohort (*n* = 50) of MOG-IgG-positive patients reported in part 1 [3] and part 2 [17] of this series, suggesting a relatively high frequency (30 %) of brainstem involvement among patients with MOG-IgG-associated myelitis and/or ON. Similarly, AQP4-IgG-positive NMO, also initially thought to affect mainly the optic nerves and spinal cord, was later reported to involve the brainstem in up to one third of all cases [16, 20, 27, 28]. We detected MOG-IgG by means of new-generation cell-based assays (CBA) employing recombinant full-length human MOG instead of enzyme-linked immunoassays, which are prone to both false-negative and false-positive results and which are no longer recommended for routine clinical diagnosis of MOG antibodies [2]; CBAs were also used for detecting AQP4-IgG [24, 25]. It is a further potential strength of this study that all patients analyzed were of Caucasian descent. This may be important since genetic factors are thought to play a role in NMO and related disorders [29].

The cases described here underline our finding from part 2 [17] that MOG-IgG-related CNS autoimmunity is not mostly a mild and monophasic disease, as suggested by some earlier studies with smaller sample sizes and shorter observation periods [4, 6], but can in fact take a relapsing and severe disease course with potentially life-threatening complications: brainstem involvement caused respiratory impairment in two patients in our series and was fatal in one of them. Respiratory insufficiency due to brainstem inflammation has previously been identified as the main cause of death in AQP4-IgG-positive NMO [30].

Besides central hypoventilation, the patients’ symptoms included dysarthria, dysphagia, impaired cough reflex, sensory impairment due to trigeminal nerve damage, facial nerve palsy, trigeminal hypesthesia, oculomotor nerve palsy and diplopia, nystagmus, internuclear ophthalmoplegia (INO), vertigo, hearing loss, balance difficulties, gait and limb ataxia, and, of particular note, intractable nausea and vomiting (INV).

### Brainstem symptoms included intractable nausea and vomiting

INV in patients with brainstem encephalitis is caused by lesions in the dorsal medulla oblongata (area postrema). While presence of an area postrema syndrome (APS) has hitherto been considered to have high predictive value for a diagnosis of AQP4-IgG-positive NMO [15, 31–35], our study demonstrates that area postrema lesions and INV also occur in MOG-IgG-positive patients. However, this is not totally unexpected. First, MOG is expressed throughout the entire CNS and, accordingly, inflammatory lesions were found in almost all areas of the CNS in our cohort [17]; there is *a priori* no reason why the area postrema in particular should be spared. Second, it has been speculated that the circumventricular organs, including the area postrema, which lack a proper blood-brain barrier (BBB), may be sites prone to entry of AQP4-IgG into the CNS; assuming MOG-IgG are pathogenic (as suggested by recent studies [2, 14]), this would also be relevant for MOG-IgG.

### Inflammation occurred in all areas of the brainstem

Apart from the pons, which was affected in 11/13 (84.6 %) patients, MRI brainstem lesions were most commonly located in the medulla oblongata (8/14 or 57.1 %), in the mesencephalon (lesions in the cerebral peduncles in 2/14 or 14.3 %), and in the cerebellar peduncles (5/14 or 35.7 %).

### Additional involvement of the diencephalon and the cerebellum

In several cases brain involvement on MRI was not restricted to the brainstem. Concomitant diencephalic (pulvinar) and cerebellar lesions – and thus rhombencephalitis – were present in 2/13 (15.4 %) and 1/14 (7.1 %), respectively. In case 3, an additional lesion was present in the cerebellum which was accompanied by limb ataxia and marked gait ataxia. Cerebellar gait and stance ataxia was also noted in case 2 as well as cerebellar dysarthria. Similarly, patient 9 in part 2 [17] presented with a (clinically silent) lesion in the cerebellar white matter; however, that patient had no lesions in the brainstem. Cerebellar symptoms have very rarely been described in AQP4-IgG-positive patients, too [20]. However, not all patients with ataxia had cerebellar lesions; some patients in this and in the total cohort [17] presented with sensory ataxia and/or unsteady gait due to paresis caused by acute myelitis.

Supratentorial brain lesions were present in 10/15 (66.7 %) patients. Lesions were found in the deep (including the periventricular) white matter (including in the corona radiata), the sub-/juxtacortical white matter (including in the insula), and, in a single patient, to some extent the cerebral cortex; furthermore, in the corpus callosum and, in one case, the basal ganglia and thalami (see part 2 of this series for exemplary MRI images [17]). In patient 3, brain and brainstem lesions were extensive and even resulted in impaired consciousness. Similarly, extensive confluent supratentorial lesions were seen in patient 7. Barkhof’s MRI criteria for MS were met in 4/15 (26.7 %) patients.

### Acute brainstem involvement was associated with ON and/or myelitis in most cases

Isolated attacks of brainstem encephalitis, i.e., attacks that were not accompanied by clinical symptoms of ON or myelitis, were very rare in the total cohort of 50 MOG-IgG-positive patients [17], accounting for only 5/276 (1.8 %) documented attacks. However, they were relatively common if only patients with a history of at least one brainstem attack are taken into account (5/21 [23.8 %] attacks, 4/15 [26.7 %] patients).

In most patients, however, brainstem attacks were at least once associated with clinical myelitis (13/15 [86.7 %] patients), ON (6/15 [40 %]) and/or supratentorial encephalitis. In 5 out of 14 patients (36 %) with a history of simultaneous brainstem encephalitis and myelitis and available MRI data, lesions contiguously stretched from the medulla oblongata into the cervical cord at least once, similar to what can be seen in AQP4-IgG-positive patients. In case 2, inflammation affected almost the entire neuraxis, including the lumbar, thoracic, and cervical spinal cord as well as areas in the brainstem and in the supraventricular white matter. This was associated with exceptionally high MOG-IgG serum titers (1:10,240) and presence of detectable MOG-IgG levels also in the CSF.

### Some patients met Wingerchuk’s criteria for NMO(SD)

At last follow-up, 12/15 (80 %) patients with brainstem encephalitis had a history of both ON and myelitis. Two patients had a history of myelitis but not of ON, and one had a history of recurrent ON (rON) but not of myelitis. All but 2 (86.7 %) had a relapsing disease course at last follow-up. Of those 12 patients, 8 (66.7 %) met the 2006 Wingerchuk criteria for NMO [36]; in the remaining 4 patients, the criteria were not fulfilled due to the presence of brain MRI lesions at onset meeting Paty’s criteria and/or because criteria for LETM were not met. On the understanding that MOG-IgG-positive ON and/or myelitis are not considered “alternative diagnoses”, i.e., based solely on clinicoradiologic findings, 9/15 (60 %) met the revised 2015 consensus criteria for NMOSD.

### Brainstem involvement may be asymptomatic

While overall there was a high degree of agreement between MRI findings and clinical presentation, in 5/15 (33.3 %) patients asymptomatic brainstem lesions were detected by MRI at least once, suggesting that subclinical brainstem encephalitis is not uncommon in MOG-IgG-related CNS autoimmunity. The true prevalence of brainstem involvement in MOG-IgG-positive patients may thus be higher than expected based solely on clinical presentation. Similarly, evidence for subclinical involvement of the brain, the spinal cord, or the optic nerves, as detected by MRI or electrophysiology, has been found in some of our MOG-IgG-positive patients (see part 2 of this series for details [17]).

### CSF and MRI findings may mimic infectious CNS disease

In one case, leptomeningeal contrast enhancement was noted at the time of disease onset. This is of particular interest in the light of recent studies indicating the existence of a lymphatic system of the CNS within the meninges [37]. Meningeal involvement has been reported in neuromyelitis optica [38–43], MS, and other disorders of putative autoimmune etiology and may indicate a new path for immune cell entry into the CNS. Moreover, this finding is of potential differential diagnostic relevance, since it may wrongly suggest acute infectious meningitis, all the more as CSF findings compatible with an infectious etiology were found in some patients: CSF pleocytosis was present in 11/14 (78.6 %) patients (50/μl, range 8-360) with available data and comprised neutrophil granulocytes in 7/11 (63.6 %) (accounting for 3-34 % of all white CSF cells) or eosinophil granulocytes in 2/6 (33.3 %), increased lactate levels in 2/3 (66.7 %) patients, BCSFB dysfunction in 5/12 [41.7 %], and CSF-restricted OCB, a mainstay of classical MS, were absent in 10/14 (71.4 %). Together with fever and other signs of systemic infection – disease onset was preceded by acute infections in at least three patients (purulent tonsillitis, ENT infection, and bronchopulmonary infection, respectively) – these CSF findings are compatible with early bacterial or viral meningoencephalitis and thus bear the risk of delayed diagnosis of autoimmune encephalitis, as was the case in one of the patients reported here. Neutrophilic and eosinophilic pleocytosis [20, 44, 45], elevated lactate levels [45, 46], and missing OCB [45, 47] are also features of AQP4-IgG-positive NMO. Elevated neutrophil counts have been previously reported also in MOG-IgG-positive pediatric patients [48]. Moreover, attacks are preceded by acute infection in 20-30 % of cases of AQP4-IgG-positive NMO [20, 49, 50], suggesting that infection-related immunologic changes or infection-related BBB disruption [45, 47] may trigger disease activity.

### Post-infectious onset and the role of BBB damage

In one of the three patients with post-infectious onset (case 6), interruption of long-term MTX treatment for RA due to infection was followed by the first attack a few weeks later, leaving the possibility that MOG-IgG were already present before clinical onset but were able to enter the CNS to a pathologically relevant degree only after infection-related BBB breakdown. In fact, a markedly elevated QAlb as well as Gd entry into the lesion was noted during acute brainstem encephalitis in that case. In AQP4-IgG-positive NMO, the autoantibody has indeed been retrospectively demonstrated in samples obtained months or years before disease onset [51–53]. Overall, 8/9 patients showed Gd enhancement during disease activity, and 3/7, including the single patient without Gd enhancement, had an increased QAlb, indicating possible BBB disruption. Primary or secondary impairment of the BBB function may be an important step in the pathogenesis of MOG-IgG-positive brainstem encephalitis, since it may allow MOG-IgG to enter the CNS.

In one patient, the first symptoms developed within 2 weeks after vaccination for diphtheria, tetanus, pertussis, polio, and influenza, and thus within a time window considered to be compatible with a post-vaccination reaction. Of note, we describe a second adult patient with disease onset shortly after vaccination against diphtheria, tetanus and pertussis in part 2 of this series [17]. The association of MOG-IgG seropositivity with infection and vaccination deserves to be investigated in more detail. MOG-IgG have also been reported in children with acute disseminated encephalomyelitis [1], a condition with suspected post-infectious or post-vaccinal etiology [54, 55]. Postvaccinal onset of NMO has been described also in a few AQP4-IgG-positive patients, although a causal link has not yet been proven [56, 57].

### MOG-IgG positivity associated with a mature teratoma

Both MOG-IgG-positive encephalomyelitis and AQP4-IgG-positive NMOSD are not usually found in a paraneoplastic context. It is therefore of potential interest that one of the patients described above had a history of mature teratoma that was removed just 2 months before onset of her CNS disorder. In addition, a ganglioneuroma was detected after disease onset in the same patient. It remains unknown whether this patient’s tumors and MOG-IgG seropositivity were related. Teratomas have been shown to play a role in the pathogenesis of NMDAR encephalitis, another autoantibody-related disorder of the CNS [58]. However, NMDAR antibodies were negative in our patient. While, to the best of our knowledge, the presence of MOG in teratomas has not been investigated so far, expression of CNPase, an oligodendrocyte marker, has been described in mature teratomas [59], and several reports on oligodendrogliomas arising in mature teratomas exist [60–63]. Ectopic expression of MOG by the patient’s tumor therefore cannot be completely ruled out.

### Influence of age and sex

The median age at first clinical brainstem attack was 31 years (range 18-53 years) in the present, adult cohort. This did not differ from the age of disease onset in the total MOG cohort (see part 2 [17]) (31 years, range 6-70; *N* = 50). Remarkably, in 7/15 (46.7 %) cases brainstem inflammation (as evidenced clinically or by MRI) was already present at or very shortly after disease onset. In the remainder, the median time between disease onset and first clinically apparent brainstem encephalitis was just 0.8 years. However, this interval varied widely, with brainstem lesions occurring for the first time only 7, 17, and 41 years, respectively, after the first attack in three patients. Together, these findings suggest that the presence or absence of brainstem involvement is not an effect of age or disease duration.

The sex ratio in our cohort (1:2.8) did neither differ from that previously reported in AQP4-IgG-negative NMO patients with and without brainstem lesions (1:2; *n* = 27) [20] nor from that in the total MOG cohort (1:2.8; *n* = 50) [17] (but is in stark contrast to the sex ratio of ~1:9 reported in AQP4-IgG-positive patients [20]), indicating that female gender is probably not a risk factor for the development of brainstem involvement in patients with MOG-IgG-positive ON and/or myelitis.

### Good recovery from acute brainstem attacks after IVMP and/or PEX

While many patients seemed to benefit from immunotherapy, treatment responses and long-term outcomes differed markedly. Treatments applied during acute brainstem attacks (with and without concomitant ON and/or myelitis) included IVMP, oral steroids, and PEX. Overall, treatment outcome was available for 18 attacks with clinical brainstem involvement. Treatment with IVMP (with and without oral tapering) or with PEX (with or without additional IVMP) were followed by good partial recovery after 6 and 3 brainstem attacks, respectively, and by full recovery after 3 and 3 brainstem attacks, respectively. No treatment was given for the last (and subsequently fatal) attack in patient 1, which occurred during palliative care, for two brainstem attacks in case 5, which remitted spontaneously, and for the only attack in case 5 (initially considered to be of infectious origin due to granulocytic pleocytosis), which partially remitted.

PEX treatment was beneficial in patient 1 during most attacks, including three brainstem attacks and one attack in which IVMP had led only to partial recovery. Similarly, PEX also resulted in full or almost full remission when used to treat attacks with brainstem involvement in patients 6 and 8 (used in combination with IVMP), as well as in patient 9 after failure of high-dose IVMP therapy.

Importantly, however, PEX treatment could not prevent relapses 1-3 months later in case 1, including the fatal brainstem attack in that patient, as well as a relapse of ON within 2 months in patient 9. Preliminary findings from our laboratory (S.J., unpublished data) show that anti-neural autoantibodies may remain detectable or reappear soon after five to seven plasma exchanges, raising the question of whether PEX treatment may be discontinued too early in some cases. Alternatively, T cell-mediated mechanisms may play a more important role in patients who do not sufficiently respond to PEX.

Both IVMP and PEX were also not always effective when used to treat attacks other than brainstem encephalitis in this cohort and in the total cohort (see part 2 for detailed analysis [17]).

### Long-term immunotherapy did not prevent brainstem attacks in some patients

Immunosuppressive or immunomodulatory (IS/IM) drugs used in our patients included mitoxantrone, AZA, rituximab, natalizumab, IFN-beta, and IVIG. Treatment responses varied considerably inter- and intraindividually. Patient 2 suffered from serious neurologic impairment during the acute attack and during the following months, but immunotherapy with IVMP, oral steroids, PEX, and, for 12 months, IVIG was eventually followed by almost complete remission and no more attacks. Pathophysiologically, it is of interest that immunotherapy and clinical recovery were paralleled by a significant drop in MOG-IgG titers from 1:10,240 to 1:640 in this patient. By contrast, IS/IM therapy was not effective in preventing brainstem encephalitis in several other cases. Of particular note, rituximab was followed by severe clinical and radiologic deterioration with myelitis and active (yet asymptomatic) lesions in the pons and medulla oblongata within a few weeks after infusion in patient 1, which is reminiscent of the possibly BAFF-mediated deterioration reported in some NMO patients after commencement of rituximab [64]. Moreover, a massive brainstem attack with dysarthria, dysphagia, left oculomotor and facial nerve palsy and new T2 lesions in the pons, pedunculus cerebellaris, cerebral crus and medulla oblongata occurred just 4 months after the last rituximab infusion. In case 5, one brainstem attack took place while the patient was being treated with AZA, one during treatment with IFN-beta, and one 9 months after commencement of natalizumab therapy. Patient 13 developed an attack of simultaneous myelitis and brainstem encephalitis four months after the first natalizumab infusion and another one (with lesions in the medulla oblongata) while on treatment with glatiramer acetate. In case 7, a severe attack involving the brainstem, supratentorial brain, and spinal cord occurred 4 weeks after commencement of AZA treatment; similarly, patient 8 experienced several relapses, including a brainstem attack, while on treatment with AZA. Of note, 14/34 relapes (in 10/17 AZA treated patients) were documented in the total cohort that took place during the latency period of AZA (months 1-6) (see part 2 of this series [17]). Of those, around 40 % occurred in patients not co-treated with oral steroids during that period. This suggests that co-treatment should be considered during the latency period of AZA treatment in MOG-IgG-positive patients, provided contraindications have been excluded.

### Disease exacerbation after IFN-beta

In common with other patients described in part 2 of this series [17], patient 4 was initially diagnosed with MS. Accordingly, she was treated with IFN-beta-1a i.m. However, commencement of IFN-beta treatment was associated with marked disease exacerbation, characterized by new brainstem and spinal cord lesions and a new clinical attack. Similarly, patient 13 developed three attacks of myelitis and/or optic neuritis while on treatment with IFN-beta 1a i.m. or, later on, IFN-beta 1a s.c. Disease exacerbation following IFN-beta administration has also been reported in AQP4-IgG-positive NMO [65–69] and likely reflects differences in the immunopathogenesis of MS and NMO. This observation is of high potential interest, since initial misdiagnosis as classical MS – and, in consequence, mistreatment with IFN-beta – might be even more common in MOG-IgG-positive patients than in AQP4-IgG-positive patients given the high rate of brain involvement in that condition [1]. Falsely classified AQP4-IgG- and, possibly, also MOG-IgG-positive patients might account for some of the occasional IFN-beta non-responders observed in MS studies. Larger studies on the efficacy of IFN-beta in MOG-IgG-positive patients treated with this substance in the past seem warranted, as does retrospective testing for MOG-IgG of samples from IFN-beta non-responders identified in past clinical trials.

### Long-term prognosis differed widely but did not depend on brainstem damage in most cases

Cases 1 and 2 illustrate that the prognosis differs widely among MOG-IgG-positive patients: while brainstem encephalitis led to respiratory insufficiency in both patients, it was fatal in the former case and remitted almost completely in the latter. Unexpectedly, residual neurologic impairment in our patients was mostly not related to brainstem damage. The median EDSS at last follow-up in patients with a disease duration of >24 months (*n* = 12) described here was 3 and ranged between 0 and 10; only 4 patients had an EDSS >3 at last follow-up (EDSS 4, 7.5, and 10 after 123, 225, and 507 months, respectively).

### Brainstem involvement was associated with a more aggressive disease course

However, the median EDSS at last follow-up in patients with brainstem involvement and a disease duration of >24 months was still higher (median 3, range 0-10, *n* = 12) than among all patients from the total cohort [17] who had no history of clinical or subclinical brainstem involvement at last follow-up and an observation time of ≥24 months (median EDSS 2; *n* = 23; *p* < 0.04), as were the total number of attacks at last follow-up (median 7.5, range 1-27, vs. median 3, range 1-28), the number of myelitis attacks (median 2, range 0-11, vs. median 0.5, range 0-3), the proportion of patients who had experienced both attacks of ON and attacks of myelitis at last follow up (75 % vs. 41.7 %), the median annualized relapse rate (1.32 vs. 0.59, p < 0.03), and the proportion of patients with additional supratentorial brain lesions (75 % vs. 30.4 %; p < 0.02). As observation times did not differ significantly between these two subgroups (median 69.5, range 34-507, vs. median 70, range 26-394), brainstem involvement seemed be a risk factor for a more severe disease course. This has potential therapeutic implications and should be addressed in future prospective studies.

### Limitations

We acknowledge some limitations of our study. Firstly, the retrospective design is a potential limitation. However, prospective studies would be difficult to perform due to the very low prevalence of the disease. Moreover, reliable tests have become available only recently; accordingly, only retrospective long-term data are currently available. Furthermore, the number of patients included and the number of items documented in the present study were high and data loss relatively low. Secondly, the multicenter design, which was necessary given the low prevalence of the condition, could be a limitation. However, the study design also strongly reduced the risk of selection bias, which was acknowledged as a possible limitation by the authors of previous large single-center studies in the field of NMO [30, 36]. Moreover, all patients were documented at university centers providing a similar standard of tertiary care. Thirdly, we cannot fully exclude a potential referral bias, since MOG-IgG testing may have been ordered particularly in patients presenting with ON and/or myelitis based on the previous literature. It is therefore conceivable that MOG-IgG-positive patients with isolated brainstem and/or brain involvement are underrepresented in our study. Finally, from a pathophysiological point of view it is a possible limitation that we cannot formally prove that the antibody was already present at disease onset in all cases, since routine MOG-IgG testing was not available in the past. However, MOG-IgG was present already at disease onset in all patients with available data in the main cohort, as reported in part 1 of this series [3]: 2 MOG-IgG positive sera were taken within the first week (at 2 and 4 days) after disease onset, 10 within the first month (median 10 days, range 2-31), and 18 within the first 3 months (median 26 days, range 2-85). The median MOG-IgG titer at disease onset was 1:2560 (range 160-20480; *N* = 18).

## Conclusions

In summary, our study demonstrates that brainstem involvement is common in patients with MOG-IgG-related ON and/or myelitis. Our findings do not support the notion that MOG-IgG seropositivity generally denotes a milder and usually monophasic variant of NMOSD as suggested by earlier, smaller studies with shorter observation periods. Most patients have a relapsing general disease course, and serious and potentially life-threatening complications of brainstem encephalitis such as respiratory insufficiency may occur. This needs to be kept in mind when deciding on long-term treatment, and attention should be paid to signs or symptoms of additional brainstem involvement in patients with MOG-IgG-positive ON and/or myelitis. Clinical manifestations of MOG-IgG-positive brainstem encephalitis are diverse and, notably, may include symptoms previously thought to be typical for AQP4-IgG-positive NMOSD, such as APS and INV, or of MS, such as INO. In accordance with what was observed in the total cohort [17], treatment with IVMP and/or PEX was associated with good recovery in many cases. As most MOG-IgG-positive patients develop relapses and since brainstem involvement may indicate a more aggressive disease course, prophylactic long-term treatment should be considered in patients presenting with MOG-IgG-associated brainstem encephalitis. Larger studies, which given the conditions’ relative rarity will require an international collaborative approach, are highly warranted to improve our understanding of the full clinical spectrum, acute and long-term treatment needs, and prognosis of MOG-IgG-related CNS disease and in particular MOG-IgG-associated brainstem encephalitis.

## Appendix

### Case 4 – Recurrent NMO starting with simultaneous bilateral ON and LETM followed by an area postrema syndrome; good partial recovery

In January 2012, a 30-year-old woman with no previous personal or family history of autoimmune disease developed sudden loss of vision and reduced visual field in the left eye and 2 days later in the right eye, followed by tetraparesis 2 weeks later. While brain MRI showed no abnormalities, spinal cord MRI revealed an LETM lesion extending from C2 to C7. The patient thus met Wingerchuk’s 2006 criteria for NMO [35]. LP revealed CSF pleocytosis with 122 leukocytes/μl (97 % mononuclear cells, 3 % granulocytes); OCB were not determined. Treatment with IVMP was followed by partial recovery. Four months later, a relapse of myelitis occurred in the high cervical cord, now associated with brainstem encephalitis. Symptoms included tetraparesis and INV. MRI showed a brainstem lesion adjacent to the fourth ventricle and including the area postrema, which continued into the spinal cord. Brainstem symptoms completely remitted 2 months after IVMP treatment, and spinal symptoms partially remitted with residual paresthesia and mild paraplegia (BMRC 4+) after 3 months.

The patient subsequently had recurrent attacks of isolated ON (3 × full recovery after IVMP) or isolated myelitis (2 × full recovery after IVMP, 1 × partial recovery after IVMP). Six of the eight attacks in this patient occurred during 36 months of treatment with AZA, all of them after discontinuation of co-treatment with oral steroids (given for 3 months). At last follow-up, an EDSS of 3 was documented. MOG-IgG was detected retrospectively in a sample taken during remission by means of a live-cell CBA (1:640) and was confirmed by use of a commercial fixed-cell CBA (Euroimmun). AQP4-IgG was negative. The patient had no previous personal or family history of autoimmune disease.

### Case 5 – Recurrent ON, LETM and brainstem attacks, with exacerbation after initiation of interferon-beta treatment; high relapse rate

A 34-year-old Caucasian woman with a positive family history for type 1 diabetes mellitus first presented with paraparesis due to transverse myelitis in 10/2006, which partially remitted after treatment with IVMP. Brain MRI showed asymptomatic lesions adjacent to the lateral ventricles. Five months later a second attack of transverse myelitis with paraparesis occurred, again with partial recovery after IVMP treatment; MR images from that time are unavailable. Later the same year, the patient developed a first attack of unilateral ON. LP revealed CSF pleocytosis but no CSF-restricted OCB. Administration of IVMP was followed by partial recovery. MS was suspected and treatment with intramuscular interferon beta-1a (IFN-beta) was started. However, 3 months later she experienced a brainstem attack with balance difficulties and vertigo. MRI revealed disease exacerbation with new lesions in the pons and in the medulla oblongata as well as multiple cervical and thoracic spinal cord lesions. Symptoms remitted spontaneously. In 01/2009 IFN-beta was discontinued and treatment with natalizumab initiated. During the period of natalizumab treatment (03/2009 – 08/2010), one further attack of brainstem encephalitis with impaired balance and vertigo occurred. In 06/2010, follow-up spinal MRI performed during remission demonstrated an LETM lesion extending from T2 to T6. The findings on brain MRI still did not meet the diagnostic criteria for MS. A diagnosis of relapsing NMO according to Wingerchuk’s 2006 criteria [35] was made. Over the following 75 months, the patient experienced eight further attacks of myelitis, one of ON, and one of brainstem encephalitis with balance difficulties and dizziness. Four of these episodes, including two of the myelitis attacks and the ON attack (each with full remission after high-dose steroid treatment) as well as the brainstem attack (complete spontaneous remission), occurred while the patient was being treated with AZA for 36 months. Using a live-cell CBA, low-titer MOG-IgG antibodies (1:160) were detected retrospectively in a sample taken during remission. MOG-IgG seropositivity was later confirmed in an independent commercial fixed-cell CBA (Euroimmun). In addition, low-titer serum IgA tissue transglutaminase antibodies and antinuclear antibodies (ANA) were found. Serum AQP4-IgG was negative. At last follow-up, an EDSS of 3 was documented.

### Case 6 – ON followed by post-infectious LETM and brainstem encephalitis 40 years later with poor outcome

At the age of 13 years this female patient had severe unilateral ON (visual acuity 10 %) with complete remission. No further neurologic symptoms occurred for 40 years. At age 53 she developed subacute tetraparesis and autonomic bladder and bowel dysfunction after a severe bronchopulmonary infection and a prodromal phase of diffuse tingling in all four extremities. She had been previously diagnosed with rheumatoid arthritis (RA) at the age of 43 years, and long-term treatment with weekly methotrexate (MTX) had been initiated at the age of 48 years. Due to the severe infection her immunosuppressive therapy had been stopped 4 weeks before onset of the neurologic symptoms. Initial MRI workup was inconclusive. Examination of CSF revealed mild pleocytosis with 30 cells/μl and a disturbed BCSFB function (QAlb 21.5) and no OCB. She was treated with empirical antibiotics and aciclovir. As the clinical presentation worsened, the patient became bedridden and lost bladder and bowel control. MRI was repeated and now revealed a longitudinally extensive spinal cord lesion extending centrally from the medulla oblongata to the mid-thoracic level with patchy Gd enhancement; brain MRI revealed no other abnormalities. No symptoms attributable to the medulla oblongata lesion were present. High-dose steroid treatment was initiated and was followed by 5 cycles of therapeutic PEX. She was restarted on her immunosuppressive treatment with MTX and recovered rapidly but only partially. At last follow-up, 1.5 years after this episode, she is ambulatory with no motor deficits but still reports painful dysesthesia and is restricted by severe sensory gait ataxia (EDSS 6.5). Anti-MOG antibodies were retrospectively assessed and found positive in a live-cell CBA of stored serum samples obtained between steroid treatment and the commencement of therapeutic PEX (1:2560) and at a follow-up visit (1:160); both samples were also positive in the fixed-cell CBA. AQP4-antibodies were negative in several samples.

### Case 7 – Recurrent LETM and subclinical ON with extensive brainstem and brain involvement; almost full recovery

A Caucasian man experienced a first attack of myelitis with marked paraparesis in February 2012 at the age of 19; this episode remitted fully after IVMP therapy. Spinal cord MRI showed a transverse inflammatory lesion at T9/10 with swelling and Gd enhancement. Brain MRI detected no abnormalities. LP revealed CSF-restricted OCB. Another attack of myelitis with hypesthesia of both legs occurred in May 2012. MRI showed enlargement of the known thoracic lesion, now spanning from T8 to T12, again with swelling and Gd enhancement. At that time, prolonged P100 latency (but with normal amplitudes) was noted on the left side (visual acuity [VA] 0.95 in both eyes). IVMP therapy was again followed by complete remission. AQP4 antibodies were negative, but NMO was suspected and, therefore, treatment with AZA started. A few weeks later, the patient developed severe headache, diplopia, and gait ataxia. MRI performed several times over a period of 4 weeks showed evolving confluent T2-hyperintense lesions in the right temporal lobe, lesions in the periaqueductal grey, the pulvinar bilaterally, and the ventral pons, as well as cervical lesions at C6/7 and an LETM lesion extending from T8 to the conus with Gd enhancement. LP revealed an elevated cell count (22/μl), total CSF protein of 460 mg/l, and positive OCB. VEP showed increased P100 latency on both sides and normal amplitudes. The clinical deficits remitted only partially after one course of IVMP and after therapeutic PEX (five exchanges). B-cell-depleting therapy with rituximab was started in August 2012 and was followed by further slow resolution of symptoms. In May 2013, after repopulation of B cells, mild ON in the right eye occurred but remitted spontaneously. Rituximab therapy was resumed and the patient remained free of disease activity with minor deficits (EDSS of 1.0) until last follow-up in December 2014. MOG-IgG were retrospectively tested using a live-cell CBA and were positive at a titer of 1:1280.

### Case 8 – Post-vaccination myelitis and recurrent ON with brainstem and brain involvement; full recovery

A 19-year-old male patient presented with headache, meningism, and photophobia. The patient had received a vaccination (diphtheria, tetanus, pertussis, polio; and influenza) 2 weeks before symptom onset. Examination of the CSF revealed pleocytosis (43 cells/μl; predominantly lymphomonocytic, 3.1 % neutrophils). Brain MRI demonstrated multiple T2 hyperintensities in the pulvinar thalami bilaterally and in the rostral putamen as well as leptomeningeal contrast enhancement. Spinal MRI revealed a lesion at C1 and another one extending from T11 to T12. Pragmatic treatment with ceftriaxone, ampicillin, and acyclovir was started, together with IVMP. After clinical recovery, the patient was discharged to a rehabilitation facility with the diagnosis of suspected viral meningoencephalomyelitis of undetermined origin. However, 1 month later he was readmitted with disorientation, headache, meningism, fever, and fatigue. CSF analysis again revealed pleocytosis (60 white cells/μL; 26 % neutrophils). Brain MRI demonstrated T2-hyperintense lesions in the midbrain, pons, thalamus, basal ganglia, and corpus callosum. Lesions involved the periependymal surfaces of the third ventricle. Spinal cord MRI showed a contrast-enhancing lesion at C5. Shortly thereafter, the patient developed proximal spastic paraparesis, urinary retention, and bilateral visual deficits, accompanied by delayed P100 latencies, MRI-detectable lesions involving the optic chiasm and both optic tracts, and a new T2 hyperintensity at C3. MOG-IgG were positive at a titer of 1:2560. AQP4 antibodies were negative. Treatment with IVMP (1 g/d for 5 days) followed by 5 cycles of PEX led to full clinical and neuroradiologic recovery within 3 months. Long-term immunosuppressive treatment with AZA was initiated. Within the following 20 months, four further attacks of ON (ranging from mild to severe) and presumably one further attack of brainstem encephalitis with hearing loss occurred, with each showing full remission after high-dose steroid treatment (EDSS 0 at last follow-up).

### Case 9 – Recurrent ON with subclinical brainstem and spinal cord involvement; almost full recovery

In December 2014, 4 weeks after an ENT infection, a 50-year-old Caucasian man developed a first, left-sided ON (blurred vision, retro-orbital pain, VA OS 30 %, P100 OS delayed) which remitted almost completely after treatment with IVMP. Neurologic examination was otherwise unrevealing except for slightly increased tendon reflexes, in particular of the lower extremities. Brain MRI showed an asymptomatic, median pontine lesion without Gd enhancement; in addition, there was inflammation of the entire optic nerve and parts of its sheath, with extensive Gd enhancement and subtle swelling compared with the other side. Spinal cord MRI demonstrated a possible lesion at C6/7. LP was unrevealing (negative OCB, no pleocytosis, no BCSFB dysfunction). Infectious causes of ON were excluded. Serologically, there was no indication of vasculitis. MOG-IgG antibodies were found to be positive at that time. Due to flaring up of the ON shortly after, the pulse therapy was repeated with 5 × 2 g IVMP, which had no effect. By contrast, PEX treatment (8 cycles) starting 10 days after the last IVMP dose resulted in almost complete recovery.

However, 2 months later a relapse of unilateral ON of the left eye occurred with complete visual loss (VA OS 0 %; Snellen chart), impaired color vision, and retro-orbital pain. Brain MRI showed inflammation of the posterior (pre-chiasmal) part of the optic nerve with Gd enhancement but no significant swelling. Standard VEP findings had worsened (P100 not detectable in left eye); flash VEP revealed a marked P100 prolongation and reduced amplitudes in the left eye. While the pontine lesion had markedly decreased in size, several new dorsolateral and central spinal cord lesions without Gd enhancement had developed (T7/8, T8/9 and T11/12). MOG-IgG were positive at a titer of 1:10,240. PEX treatment (8 cycles) led to almost full clinical recovery, with discretely disturbed color vision and an increased sensitivity to glare as residual symptoms. Standard VEP also improved following PEX treatment, but still showed a delay in latency (159 ms) and reduced amplitudes compared with the right eye. Treatment with rituximab was commenced without complications. At last follow-up (May 2015) the patient had almost fully recovered (VA OS 0.9; EDSS 1.5).

### Case 10 – Protracted and recurrent ON with complete visual loss and signs of mild myelitis and brainstem encephalitis; complete recovery

A previously healthy 37-year-old man presented with a 4-day history of bifrontal headache, pain upon eye movement, and bilateral complete visual loss and color desaturation. Ophthalmoscopy revealed bilateral papilledema. Markedly prolonged P100 latencies were noted in both eyes, suggestive of demyelination. Brain MRI showed a bilateral optic nerve lesion with swelling and Gd enhancement but did not reveal any brain lesions. Spinal cord MRI was normal. Serum AQP4-IgG was negative, as were CRP, ANA, ANCA, rheumatoid factor, lupus coagulant, anti-gliadin and anti-transglutaminase, HIV, HTLV-1, and vitamin B12. CSF examination demonstrated mild pleocytosis (5 cells/μl; including 34 % neutrophils and 6 % eosinophils) and identical OCB in CSF and serum but was otherwise normal (including a negative MRZ reaction). Treatment with IVMP (1 g/d for 5 days) was followed by marked improvement in VA. However, only 4 days later the patient was readmitted with new visual impairment; scotoma; hypesthesia of the right lower face and nose, the right hand, and the lateral parts of the right arm; paresthesia in the fingers of both hands and in both legs; and headache. No repeat MRI was performed. VEP and SSEP were normal. AQP4-IgG was still negative. Repeat LP revealed a mild lymphomonocytic pleocytosis (8 cells/μl). The patient’s visual deficits increased (0.6 OS, 0.8 OD) despite a second, prolonged IVMP cycle (1 g/d for 7 days); symptoms stabilized (but did not improve) after PEX (five exchanges). Two days after discharge the patient was again readmitted with further decrease in VA in the left eye (0.3), marked bilateral scotoma (OS> > OD), headache (NAS 3), and vertigo (“like in an elevator moving down”). VEP showed a further increase in P100 latency in the left eye. Treatment with IVMP was followed by complete remission (VA 1.0 in both eyes at discharge). However, follow-up VEP still showed markedly delayed, albeit slightly improved, P100 latencies in both eyes (154 ms compared to 178 ms at previous examination). To prevent further flare-ups after steroid withdrawal, treatment with oral steroids (100 mg/d; tapering to 10 mg/day) was initiated and, taking into consideration the patient’s positive MOG-IgG serostatus, long-term treatment with AZA started. At follow-up 63 days after onset, the patient reported persisting color desaturation and impaired contrast perception, together with paresthesia in the left leg. The patient discontinued AZA after two months.

Six months after onset and one month after the first infusion of rituximab (1000 mg), he developed a relapse of ON, which fully responded to high-dose IVMP treatment, another infusion of rituximab (1000 mg) and subsequent oral steroid treatment. Another two months later, shortly after oral steroids were tapered out, a third attack of ON occurred, which was associated with mild hemiparesis and hemihypesthesia, which fully remitted following treatment with IVMP and, subsequently, oral steroids.

### Case 11 – Myelitis and recurrent ON with lesions in the cerebellar peduncle and the frontal lobe; partial remission

A previously healthy 44-year-old Caucasian woman presented in February 2011 with sensorimotor paraparesis and urinary incontinence. Spinal MRI showed a cervicothoracic, longitudinally extensive spinal cord lesion with patchy Gd enhancement and further short, patchy spinal cord lesions. Brain MRI revealed a lesion in the cerebellar peduncle and one small cerebral lesion located in the left frontal lobe with slight Gd enhancement. The myelitis symptoms partially recovered under IVMP (1 g/d for 5 days) with residual detrusor sphincter dyssynergy and slight gait ataxia. Subsequently, the patient suffered recurrent urinary tract infections, which were effectively controlled by use of methionine (3 × 500 mg/d). In May 2011 she developed vision loss in the right eye (VA 0.2) with prolonged P100 latency and reduced P100 amplitudes. VA recovered to 0.75 after IVMP therapy within two months. Two months later, the patient experienced an attack of ON in the previously unaffected left eye (VA reduced to light perception). Partial remission was achieved after 5 PEX cycles; previous treatment with IVMP for 5 days had not resulted in any improvement. In August 2011, immunosuppressive treatment with mitoxantrone was started (10 mg/m^2^; 4 cycles at 6-week intervals; no relapses). In January 2012, therapy was switched to AZA (2 mg/kg). After at least three attacks of unilateral ON under AZA, the patient’s treatment was changed to rituximab (1000 mg i.v. in February 2015 and March 2015, respectively). Complete B cell suppression was noted 3 months after treatment. In June 2015 she developed bilateral ON with VA of 0.2 in the right eye and 0.05 in the left eye. After IVMP (5 g) and five cycles of PEX, VA improved. At the time of her last follow-up visit in August 2015, VA was 0.8 in both eyes and an EDSS of 2.5 was documented.

### Case 12 – Simultaneous ON and LETM with post-partum onset and pontomedullary brainstem encephalitis; full recovery

A 27-year-old woman developed a first, painful attack of ON in July 2012, just 6 weeks after the delivery of her first child. Ophthalmologic assessment revealed VA of 0.8, papilledema, and delayed VEP latencies (but normal amplitudes) in the left eye. Cranial MRI showed swelling as well as intraneural and perineural contrast enhancement extending over the anterior two thirds of the left optic nerve but was otherwise unremarkable. The CSF had normal cell and protein profiles with negative OCB. High-dose intravenous pulse therapy with IVMP (1000 mg/d for 3 days) was followed by complete resolution of symptoms. However, a few days later (and 2 weeks after attack onset) the patient noticed right-sided ocular pain upon eye movements, diplopia, paresthesia around her mouth and her waist, and urge incontinence. On neurologic examination she had a left-sided sixth nerve palsy, gaze-directed horizontal and vertical nystagmus, and a mildly unsteady gait. VA was normal. MRI of the brain and spinal cord now revealed T2-hyperintense lesions extending from the pontomedullary junction throughout the cervical cord as far as C5 as well as an additional T2 hyperintensity located in the juxtacortical insular region on the left side. None of these abnormalities showed Gd enhancement. Repeat lumbar puncture disclosed lymphocytic pleocytosis (59 cells/μl) in the presence of normal lactate and protein levels, including negative OCB. Antineuronal and AQP4 antibodies were negative and there was no evidence of an infectious etiology. The patient was treated with IVMP (1000 mg daily for 5 days) followed by 80 mg orally with subsequent tapering for 7 weeks.

In September 2012, at a maintenance dose of 5 mg methylprednisolone per day and after improvement of symptoms, she experienced right-sided ON leading to a moderate decline in visual acuity (0.6). She received a further course of IVMP (1000 mg daily for 3 days) and oral therapy with decreasing doses of prednisolone (starting with 80 mg) for a total of 4 weeks with a permanent maintenance dose of 10 mg daily. This regimen prompted normalization of visual impairment. AQP4-IgG, tested in a CBA, was again negative. Long-term treatment with AZA and oral corticosteroids was started.

In October 2012 (6 weeks after onset of the last attack), just 4 days after termination of steroid therapy and 1 day following reduction of AZA from 150 mg to 100 mg daily because of elevated liver enzymes, the patient developed a third episode of painful ON with right-sided visual impairment. She received another course of 1000 mg IVMP daily for 5 days followed by oral tapering, which resulted in complete recovery. AZA was stopped in November 2012 and replaced by MTX in February 2013. The patient discontinued MTX in November 2014 because she wanted a second pregnancy. At that time, MRI showed mild residual signal hyperintensity in the left optic nerve, no brain lesions, and a residual short spinal T2 hyperintensity at the C4/5 level. At last follow-up in 09/2015, she had not experience new neurologic symptoms (EDSS 0). Three stored sera obtained in July 2012 were retrospectively tested positive for MOG-IgG at a titer of 1:5120 each.

### Case 13 – Recurrent ON and myelitis; hypesthesia of the tongue and cheek; lesions in the medulla oblongata and in the pons; ongoing disease activity during treatment with IFN-beta and GLAT

A 19-year old woman developed acute hypesthesia of both hands and Lhermitte’s sign in 2007. Fifteen months before that event, she had suffered from an episode of extremely painful headache starting 4 weeks after influenza vaccination and lasting for 14 days (no previous history of headache). Brain MRI was normal at the time of attack onset, but spinal cord MRI revealed a single lesion at C2. CSF examination demonstrated lymphocytic pleocytosis (33 cells/μl) and intrathecal IgG and IgM synthesis. VEP, SSEP and MEP were normal. Two months later, she developed unilateral ON. Both attacks were treated with IVMP, which was followed by complete recovery in each case. Over the following 8 years, she developed at least seven more attacks of myelitis and two more attacks of ON despite immunomodulatory or immunosuppressive treatment with IFN-beta 1a s.c., IFN-beta 1a i.m., GLAT, natalizumab or fingolimod for suspected MS, with partial or full recovery following steroid treatment. While most myelitis attacks were characterized by sensory symptoms (including girdle-like or, later, generalized dysesthesia and pain), some resulted in spastic paresis of the lower extremities and impaired ambulation requiring unilateral assistance. Repeat MRI demonstrated both short spinal cord lesions and LETM lesions, partly with swelling of the spinal cord and contrast enhancement. ON attacks were mostly mild, but severe attacks occurred as well (VA 0.25, peripheral scotomas). At least twice, she experienced simultaneous ON and myelitis. Several repeat brain MRIs showed (partly contrast-enhancing) juxtacortical, deep white matter, callosal, and periventricular lesions. While the initial LP had revealed quantitative evidence for intrathecal synthesis of both IgG (33 % of total CSF IgG) and IgM (59 % of total CSF IgM), only intrathecal IgG synthesis was detected at repeat LP during another acute an acute attack 10 months later. Nineteen months after onset, she developed an attack of myelitis with hypesthesia in both legs, Lhermitte’s sign, and lesions at C2, C6, and C7 which was accompanied by hypesthesia of the tongue and facial hypesthesia, dysesthesia and pain, suggesting brainstem encephalitis. However, no brainstem MRI was performed at that time. Two follow-up MRIs showed T2-hyperintense, non-contrast enhancing lesions in the medulla oblongata and in the pons, which remained detectable over a period of at least once year. Additional symptoms attributable to brainstem lesions included impaired coordination, impaired ambulation, and disturbed smooth pursuit. Microbiological examinations for *Borrelia burgdoferi*, HIV, HBV, HCV and VZV as well as serological examinations for rheumatic diseases were negative. At last follow-up in 2016, an EDSS score of 3.5 was documented.

### Case 14 – LETM with pontomedullary brainstem encephalitis and relapsing ON; almost full recovery

A female Caucasian patient developed a first attack of unilateral ON at the age of 17 but recovered fully after IVMP treatment. Brain MRI was normal at onset. CSF examination revealed mild pleocytosis (8 cells/μl) and evidence of BCSFB dysfunction but not of intrathecal IgG synthesis. Seven months later, she developed paraparesis due to an attack of acute myelitis; IVMP treatment was followed by complete recovery. Over the next 56 months, five further attacks of ON occurred (three while on GLAT for suspected MS, one with transient unilateral blindness several weeks after GLAT had been discontinued due to an unplanned pregnancy, and one after 8 months of AZA treatment), all of which responded to IVMP therapy, as well as two further attacks of non-longitudinally extensive transverse myelitis, one of which was associated with internuclear ophthalmoplegia (INO). The latter attack occurred under treatment with IVIG. Previously, AZA had had to be discontinued due to an increase in liver enzymes and a planned pregnancy. During that attack, brain MRI showed a large, Gd-enhancing brainstem lesion affecting the pons bilaterally, both pedunculi cerebelli, and the paramedian pontomedullary junction. Spinal cord MRI showed two separate lesions at C2/3 and C5. In addition, Gd-enhancing supratentorial lesions located peritrigonally and in the corona radiata were noted. PEX was required in addition to IVMP to achieve remission of symptoms. Treatment with rituximab had to be stopped after the first infusion due to an allergic skin reaction. Under subsequent immunosuppression with ofatumumab (18 months so far, 4 cycles) one additional ON attack has occurred (14 months after commencement of therapy). Despite the high number of nine ON attacks and complete blindness during one of those attacks, the patient had almost normal VA (0.9) at last FU. VEP showed prolonged P100 latencies and reduced amplitudes.

### Case 15 – Hemihypesthesia including the face; bilateral lesion in the pons and the cerebellar peduncles; myelitis; partial recovery

This 25-year-old Caucasian woman first presented in February 2016 with tingling paresthesias of her left arm and leg, which had developed over the course of 2 days. She had a history of asthma bronchiale and neurodermitis, but except for a bipolar disorder and rare attacks of typical migraine no history of neurological symptoms. She had suffered from fever and fatigue 2 weeks before her neurological symptoms started. Neurological examination revealed paramedian moderate left-sided tactile hemihypesthesia including the face and a pathologically increased left knee-jerk without other pyramidal signs, as well as an unstable Romberg stance with eyes closed, but was otherwise unremarkable. In particular she had no limb ataxia and no further cerebellar signs. Autoimmune serology revealed a strongly increased ANA titer of 1:320 with a speckled/spindle apparatus staining pattern, while no ENA antibodies and no ANCA were detectable. Cranial MRI revealed bilateral lesions of the pons, more marked on the right side, and bilateral lesions in the cerebellar peduncles as well as a single small lesion directly adjacent to the left lateral ventricle, while MRI of the whole spinal cord showed no abnormalities. CSF analysis revealed pleocytosis (150 white cells/μl; 29 % lymphocytes, 45 % monocytes, 26 % granulocytes), total protein of 56.1 mg/dl (<50), and an increased albumin quotient of 11.3 (upper limit of age-corrected normal = 7.0); isoelectric focusing revealed identical OCB in serum and CSF; no intrathecal antibody synthesis against measles, rubella, varicella zoster, and herpes simplex virus was detectable. CSF PCR analyses of a panel of neurotropic viruses were negative. Serology of serum and CSF was negative for Lyme disease and syphilis. Visual, sensory, and motor evoked potentials were normal. Owing to the history of recent fever and CSF pleocytosis including neutrophils, treatment with aciclovir i. v. and ceftriaxone was started, based on a tentative diagnosis of infectious, primarily viral encephalitis on the day of admission. Aciclovir was discontinued after negative viral PCR results became available, while ceftriaxone was continued for 14 days. After an initial worsening of the patient’s hemihypesthesia, occurrence of a mild left-sided hemiparesis, and newly occurring intermittent urinary hesitancy over a few days, the symptoms started to gradually improve. Another lumbar puncture 14 days after admission revealed a cell count of 98/μl. The patient was discharged with partial relief of symptoms. Positive anti-MOG IgG and negative aquaporin-4 antibody results arrived after discharge.

Four weeks later the patient was again admitted to our department with new sensory disturbances affecting her trunk and legs below T10 and stance and gait ataxia. MRI now revealed a lateral spinal cord lesion spanning from T8 to T9 without contrast enhancement. Another spinal tap showed pleocytosis (20 white cells/μl), a now normal albumin quotient of 5.6, and again identical OCB in serum and CSF. Flow cytometric CSF analysis revealed 10 % lymphocytes, all CD3-positive, and a marginally increased CD4/CD8 ratio of 4.4. Visual and sensory evoked potentials were again normal, while the patient rejected analysis of motor evoked potentials. Treatment with IVMP (5 × 1 g/day) induced partial remission of her new symptoms. Cranial MRI after steroid initiation revealed moderate regression of all known brain lesions and no new lesions. After a well-tolerated test dose, secondary prophylactic treatment with azathioprine 150 mg/day was started.

The patient next attended our neuroimmunological outpatient clinic for a routine follow-up appointment in June 2016. She reported that the residual left-sided sensorimotor symptoms from her first attack had intensified in the previous 4 weeks, indicating a potential mild relapse. Two weeks before the beginning of this intensification of symptoms she had had a feverish respiratory infection, for which she had received antibiotic treatment. Based on a peripheral blood lymphocyte count of 2470/μl, the azathioprine dose was increased. An EDSS of 3 was documented.

## Abbreviations

AQP4: aquaporin-4
AZA: azathioprine
BCSFB: blood-CSF barrier
BMRC: British Medical Research Council
CSF: cerebrospinal fluid
EDSS: expanded disability status scale
GLAT: glatiramer acetate
IFN-beta: interferon-beta
IgG: immunoglobulin G
IM: immunomodulatory
IS: immunosuppressive
IVIG: intravenous immunoglobulins
IVMP: intravenous methylprednisolone
LETM: longitudinally extensive transverse myelitis
LP: lumbar puncture
MOG: myelin oligodendrocyte glycoprotein
MRI: magnetic resonance imaging
MS: multiple sclerosis
MTX: methotrexate
NMO: neuromyelitis optica
NMOSD: neuromyelitis optica spectrum disorder
OCB: oligoclonal bands
ON: optic neuritis
QAlb: albumin CSF/serum quotient
RA: rheumatoid arthritis
SSEP: somatosensory evoked potentials
VA: visual acuity
VEP: visual evoked potentials
